# The roles of multifunctional protein ErbB3 binding protein 1 (EBP1) isoforms from development to disease

**DOI:** 10.1038/s12276-020-0476-z

**Published:** 2020-07-27

**Authors:** Inwoo Hwang, Hyo Rim Ko, Jee-Yin Ahn

**Affiliations:** 1 Department of Molecular Cell Biology, Sungkyunkwan University School of Medicine, Suwon, 16419 Korea; 2 Single Cell Network Research Center, Sungkyunkwan University School of Medicine, Suwon, 16419 Korea; 3 Samsung Biomedical Research Institute, Samsung Medical Center, Seoul, 06351 Korea

**Keywords:** Molecular biology, Growth factor signalling

## Abstract

The roles of the two isoforms of ErbB3-binding protein 1 (Ebp1) in cellular function and its regulation in disease and development is a stimulating area in current fields of biology, such as neuroscience, cancer biology, and structural biology. Over the last two decades, a growing body of studies suggests have suggested different functions for the EBP1 isoforms in various cancers, along with their specific binding partners in the ubiquitin-proteasome system. Owing to the specific cellular context or spatial/temporal expression of the EBP1 isoforms, either transcriptional repression or the activation function of EBP1 has been proposed, and epigenetic regulation by p48 EBP1 has also been observed during in the embryo development, including in brain development and neurologic disorders, such as schizophrenia, in using an Ebp1 knockout mouse model. Here, we review recent findings that have shaped our current understanding of the emerging function of EBP1 isoforms in cellular events and gene expression, from development to disease.

## Cell biology: deciphering differences between “sibling” proteins

A pair of proteins that originate from a common gene exert strikingly different effects on embryonic development as well as tumor growth and progression. RNA transcripts generated from the *PA2G4* gene can undergo enzymatic processing to yield two different protein products, p42 EB1 and p48 EB1. These proteins differ by the presence or absence of 54 amino acids at one end, and Jee-Yin Ahn at the Sungkyunkwan University School of Medicine, Suwon, South Korea, and colleagues have reviewed current insights into the functional consequences of this difference. The two proteins bind to distinct sets of molecular partners. The p48 form appears to regulate a host of genes involved in brain development, but also appears to drive cancerous growth in various tumors. In contrast, p42 is scarcer during development, and appears to inhibit tumor formation.

## Introduction

ErbB3-binding protein 1 (Ebp1) is a member of the PA2G4 family of proteins, which regulate proliferation^1^, and it is an evolutionarily conserved, ubiquitously expressed, and multifunctional protein. The *Ebp1* gene (*PA2G4*) is composed of ten exons and encodes two splice variants, p48 EBP1 and p42 EBP1 (lacking 54 amino acids at the N-terminus)^2^. The difference in the N-terminus between p48 and p42 EBP1 directs unique function and association with different binding partners and their regulation^3^. The longer form of p48 Ebp1 is the predominant form that is highly expressed in mammalian cells during embryogenesis and is maintained at a certain level after birth. p42 EBP1 can be detected after birth when the p48 EBP1 level is decreased relative to its level at the embryonic period^4^. In several types of cancer cells, including glioblastoma multiforme (GBM) and lung cancer cells, p48 EBP1 expression was notably high, which was noteworthy because of its oncogenic function. p48 EBP1 enhances cancerous growth and cell cycle progression and prevents cell death through the regulation of HDM2 activity for p53^5–7^. Conversely, p42 EBP1 is barely detectable in cancer cells, and its ubiquitin-proteasome system (UPS)-dependent degradation acts as a tumor suppressor. This leads to the UPS-dependent degradation of the p85 subunit of PI3K, inhibiting its activity^8,9^.

The X-ray structure of p48 EBP1 confirms the presence of a methionine aminopeptidase fold; it has also been highlighted that p42 EBP1 is missing 1.5 helices at the amino terminus^10^. Cryo-EM reconstruction studies have suggested that EBP1 can potently regulate translation as it binds to DNA^11^ and an array of RNA targets, including FMDV-IRES^12^, the 3′-UTR region of bcl2 mRNA^13^, various RNAs^10,14^, and the mature human 80S ribosome^15^. However, such data cannot address the specific roles of EBP1 in translational regulation. In addition, although experimental validation is lacking, p42 EBP1 is assumed to not bind to ribosomes when it lacks the 54 amino acids that are involved in recognition of the cs1 element of ES27L-B^15^. Nonetheless, studies suggest that two of the EBP1 isoforms could have different properties in terms of overall conformation, folding, and differential interacting partners, including ribosome binding.

Recent studies have proposed a potent role for EBP1 in embryonic development. For instance DPPA4 expression is restricted to early embryos and diminishes at E13.5. Furthermore, it interacts with p48 EBP1 in pluripotent stem cells yet not in healthy, nonpluripotent stem cells; further, this interaction was reduced upon differentiation of pluripotent stem cells^16^. Overexpression of EBP1 disrupts muscle progenitors, leading to neurogenic-like states. EBP1 protein is healthily expressed in myoblasts during development and regenerative myogenesis. This expression controls the balance between proliferation and differentiation in the chick embryo model^17^. In *Xenopus*, loss of Ebp1 leads to downregulation of the neural border zone, neural crest, and cranial placode genes by interacting with the transcription factor Six1^18^. The only *Ebp1* knockout mouse, which was generated to study embryonic development, came from our lab, and it showed that embryos of *Ebp1*^(−/−)^ mice exhibited developmental abnormalities, including brain malformation and severe hemorrhage development with massive cell death and failure in cell cycle progression^19^. Notably, we suggest that an essential role for EBP1 in development is the repression of the gene-silencing unit Suv39H1/DNMT1, with the suppression here leading to target gene expression.

**Fig. 1:**
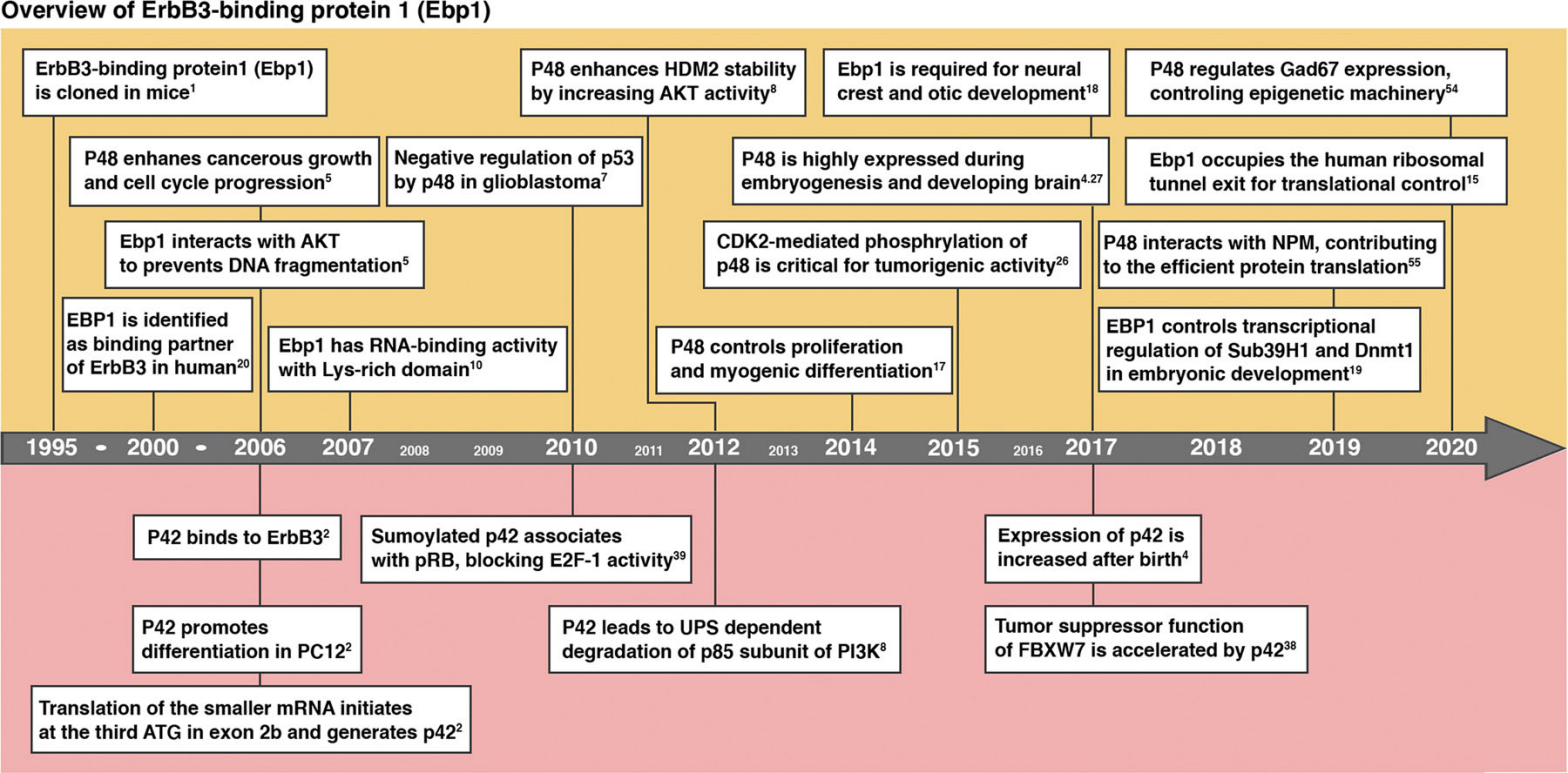
Overview of ErbB3-binding protein 1. Murine p38-2G4, a homolog of human ErbB3-binding protein 1 (EBP1, PA2G4), was originally identified as a cell cycle-specifically modified and proliferation-associated nuclear protein in 1995, and it has been well defined for its novel function over two decades. The upper panel shows the findings for p48 EBP1, and the lower panel shows the findings for p42 EBP1.

The precise molecular mechanisms of how EBP1 contributes to different steps in cellular processes are not well defined, despite independent studies over the last two decades that demonstrate that EBP1 regulates various cellular events, including proliferation, survival, and differentiation, either in embryogenesis or human disease (Fig. 1). In this review, we focus on the advances made in understanding the regulatory mechanisms and molecular biology regarding EBP1 isoforms in development and disease.

## The *Ebp1* gene and protein structure of EBP1 two isoforms

The ErbB3-binding protein 1 mouse *Ebp1* gene (*Pa2g4*) (chromosome 12q13.2 in humans; chromosome 10 D3 in mouse) comprises ten exons and extends over 8.4 kb. The ATG initiation codon is located in exon 1, and the STOP codon is located in exon 10. One *Ebp1* isoform has been reported; it encodes 394 amino acids (cDNA: AK076286, protein NP_035249), although it encodes two alternatively spliced EBP1 isoforms, p48 (394 a.a.) and p42 (340 a.a.). *Pa2g4* is located ~1.3 kb downstream of *Erbb3*, and it encodes the human epidermal growth factor receptor heregulin. Furthermore, *Erbb3* overlaps with the *Ebp1* putative regulatory region. Although EBP1 was identified as a binding partner of ERBB3^20^, only p42 EBP1 binds ErbB3; p48 EBP1 did not bind to ErbB3^2^.

In humans, the two mRNA transcripts of *Ebp1* are ~2.2 and 1.7 kb in size^21,22^. Ebp1 contains three in-frame ATG codons in its N-terminus. Translation of the longer transcript initiates at the first ATG in exon 1 and generates a 394 amino acid protein, p48 EBP1. Alternative splicing of pre-mRNA presumably leads to the omission of the 29 nucleotides that contain the second ATG, resulting in a smaller transcript. The frameshift that is caused by this mutation leads to translation of the smaller mRNA, which is initiated at the third ATG in exon 2b and the generation of p42 EBP1. p48 EBP1 migrates at an apparent molecular weight of 48 kDa, while p42 EBP1 migrates at an apparent molecular weight of 42 kDa, as shown by SDS/PAGE^2^.

The amino acid M55 in p48 corresponds to M1 in p42; for simplicity, we use M55 hereafter to refer to both p42 and p48. p48 Ebp1 (394 amino acid) contains one initiator methionine (this residue is removed), two nucleolar localization signals in exon 1 (lysine 20 and 22 residues) that are necessary for nucleolar localization^14^, and a peptidase m24 domain (104–120 a.a.). The X-ray structure of EBP1 (8–360 a.a.) revealed that it possesses the predicted methionine aminopeptidase (MetAP) fold (type II); however, it did not possess catalytic activity, which may be due to its shorter N-terminus (~15- residue missing) and a C-terminal extension of 50 residues harboring a highly positively charged lysine patch. Although the lysine-rich C-terminal region was not part of the crystallization model (missing 30 C-terminal regions), the positive surface charge of EBP1 was found to cluster on the convex side and included a charged surface loop (62–72 amino acid) and C-terminal extension (338–360) that can interact with proteins and RNA^10,23^. Unlike the prediction with sequence analysis that suggests EBP1 contains two conserved RNA binding domains as a consensus sequence with a sigma 70-like motif (46–64 amino acid) and ds RNA binding domain (91–156)^14,24^, neither of these domains is observed in the crystal structure of EBP1. Rather, a lysine-rich 364–373 amino acid sequence is likely to be the key determinant for RNA binding. This was highlighted by the deletion of this region that severely impairs EBP1 binding to RNA or DNA targets and mediates the interaction with internal ribosome entry site (IRES) of the foot and mouth disease virus (FMDV), exhibiting sequence-specific stimulation of FMDV-IRES-mediated translation initiation^10^. The predicted crystal structure of p42 lacks 1.5 alpha-helices, and a large hydrophobic surface of p42 is exposed to the solvent. This exposure may cause destabilization of p42 Ebp1, accounting for its significantly lower levels of expression in many types of cancer cells^2,6,10,25^.

A recent high-tech cryo-EM reconstruction of EBP1 highlighted that EBP1 binds to the human mature 80S ribosome and occupies the exit site of the ribosome and recruits the rRNA expansion segment ES27L, which is one of the longest tentacle-like dsRNA insertions. However, the biological impact of EBP1 in the EBP1-ribosome complex is not clarified, as EBP1 binding did not alter the translational elongation cycle or any conformational changes in the ribosomal proteins and rRNA in the peptidyl transferase center^15^. Moreover, EBP1 occupancy on the ribosome was not found to be different when comparing healthy cells or cells derived from cancer cells (HEK293 cells vs. HeLa cells). Despite the cell proliferation ability of p48 EBP1 in cancer cells^6,7,26^, neither of the experiments revealed a direct effect of EBP1 on translational inhibition. This may be due to the different roles of the EBP1 isoforms in cell growth. Predictably, p42 EBP1 may have impaired binding to the ribosome, as the missing 54 amino acids are involved in recognition of ES27L and are an integral feature of the MetAP-like fold. Therefore, EBP1 binding to the ribosome is likely isoform-specific and perhaps correlates to tumor progression^15^. However, the translational inhibitory role of p48 EBP1 or its mechanistic study has not yet been experimentally validated.

## Oncogenic functions of p48 EBP1 and tumor suppressor p42 Ebp1 in human cancers

We have identified two isoforms of the EBP1 protein, p48 EBP1 and p42 EBP1, that are translated and expressed in humans, rats, and mice, but only p42 EBP1 can bind to ErbB3^2,6,26,27^. Before we reported two isoforms of EBP1^2^, early studies of EBP1 from Dr Hamburger’s group suggested that EBP1 was a binding partner of ErbB3, and the assumption was that it was p42 EBP1. A series of studies have proposed that EBP1 is a tumor suppressor in breast and prostate cancer cells following the study of p42 EBP1, in which translation starts only at the third in-frame ATG. In support of this observation, the pBK/Ebp1 expression plasmid was used throughout their study; the plasmid encoded a 372 amino acid protein lacking N-terminal 22 amino acids^26,28–33^. Human Ebp1 cDNA encodes a protein predicted to have 394 amino acids; as expected, the protein was found to have 394 amino acids when it was empirically analyzed. In agreement with the tumor-suppressive activity of EBP1, our studies also demonstrated that p42 EBP1 acts as a tumor suppressor in GBM and non-small lung cancer (NSCLC) cells^7,34^. Strikingly, p42 EBP1, and not p48 EBP1, interacts with the p85 subunit of phosphatidylinositol 3-kinase (PI3K) and couples the p85 subunit to heat shock protein 70 (HSP70). The carboxy terminus of Hsp70 interacting protein (CHIP)-mediated UPS thereby promotes a reduction of p85 subunits, consequently leading to inhibition of its lipid kinase activity in both in vitro and in vivo models^7^. Moreover, p42 EBP1 also suppressed AKT kinase activation, resulting in inhibition of tumorigenic properties of NSCLC^34^. Furthermore, in salivary adenoid cystic carcinoma^35^, the tumor suppression activity of EBP1 was demonstrated using a pcDNA-ebp1 plasmid obtained from Dr. Hamburger. This work suggests that this tumor suppression effect comes from p42 EBP1.

p42 EBP1 protein levels were notably lower in GBM patients than they were in healthy tissues obtained from the same patient, while p48 EBP1 was highly expressed in GBM and NSCLC cells^6,34^. These results are in agreement with the finding that only p42 EBP1 is polyubiquitinated and degraded in human cancer cells via hBre1^25^. Interestingly, in colorectal cancer cells, both p48 EBP1 and p42 EBP1 bind to FBXW7 (F-box and WD40 domain protein 7), a substrate recognition subunit of the SCF (SKP1/CUL1/F-box protein) E3 ligase complex, and p48 EBP1 is ubiquitinated and degraded in a GSK-3b-dependent manner, whereas p42 EBP1 does not undergo degradation. In contrast, a tumor suppressor function of FBXW7 was accelerated by p42 EBP1, enhancing the association of FBXW7 and its oncogenic targets, including c-Myc, Aurora-A, and cyclin E. At the same time, p48 EBP1 contributes to the relocalization of FBXW7 from the nucleus to the cytoplasm, where it is not able to bind to its oncogenic nuclear targets^36^. Moreover, only p42 Ebp1 is sumoylated on lysine (K) residues 93 and 298 by translocation in liposarcoma/FUS, and this process that is regulated by PKC-δ-mediated S360 phosphorylation. p42 sumoylation mediates its nucleolar translocation and is required for its stability. In turn, sumoylated p42 is associated with pRB, which thereby blocks E2F-1 transcriptional activity and inhibits cell proliferation^37^. These studies provide insight into the mechanisms underlying tumor suppressor p42 downregulation in cancer cells.

In contrast to the tumor-suppressive activity of p42 EBP1, reflecting the anti-apoptotic and growth-promoting activities of p48^2,38^, p48 EBP1 is expressed at high levels in cancer cells^25^, including GBM^6^, anaplastic large cell lymphoma^39^, colorectal cancer^40^ and acute myeloid leukemia (AML)^41^. Moreover, patients with breast cancer who express high levels of *PA2G4* have poor clinical outcomes^42^, and patients with GBM or pancreatic ductal adenocarcinoma who have high protein levels of p48 also have poor clinical outcomes^6,43^. In oral squamous cell carcinoma, Ebp1 is upregulated. It binds to the *podoplanin* promoter, which leads to a dramatic increase in both RNA and protein levels of podoplanin. Podoplanin is highly expressed in various human cancers and is associated with tumor progression as well as the pathogenesis of oral cancer^44^.

**Fig. 2:**
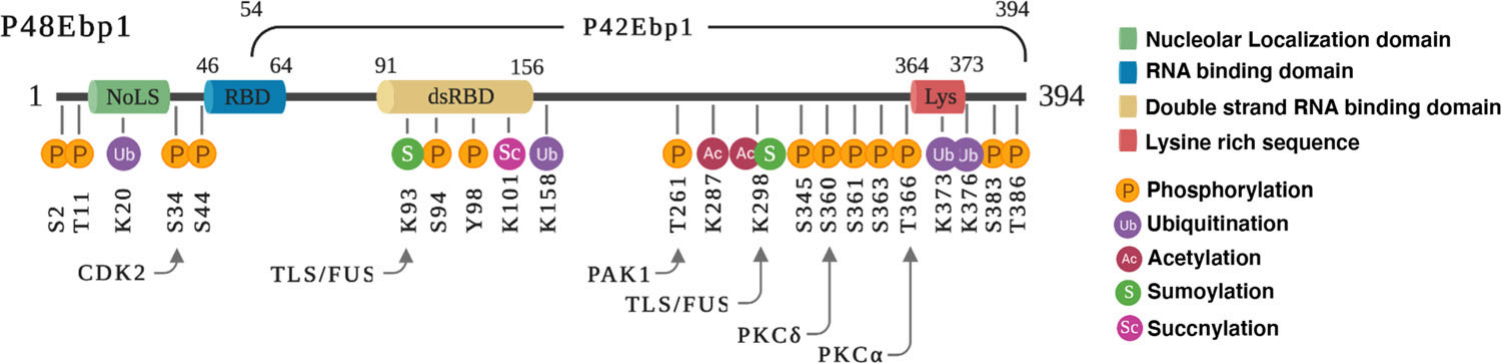
Protein structure and modifications of EBP1 isoproteins. The two EBP1 isoproteins, p48 (394 a.a, 48 kDa) and p42 (340 a.a, 42 kDa), undergo multiple post-translational modifications: Cyclin-dependent kinase 2 CDK2; Fused in sarcoma/translocated in liposarcoma TLS/FUS; p21-activated serine/threonine kinase 1 PAK1; Protein kinase C PKC.

**Fig. 3:**
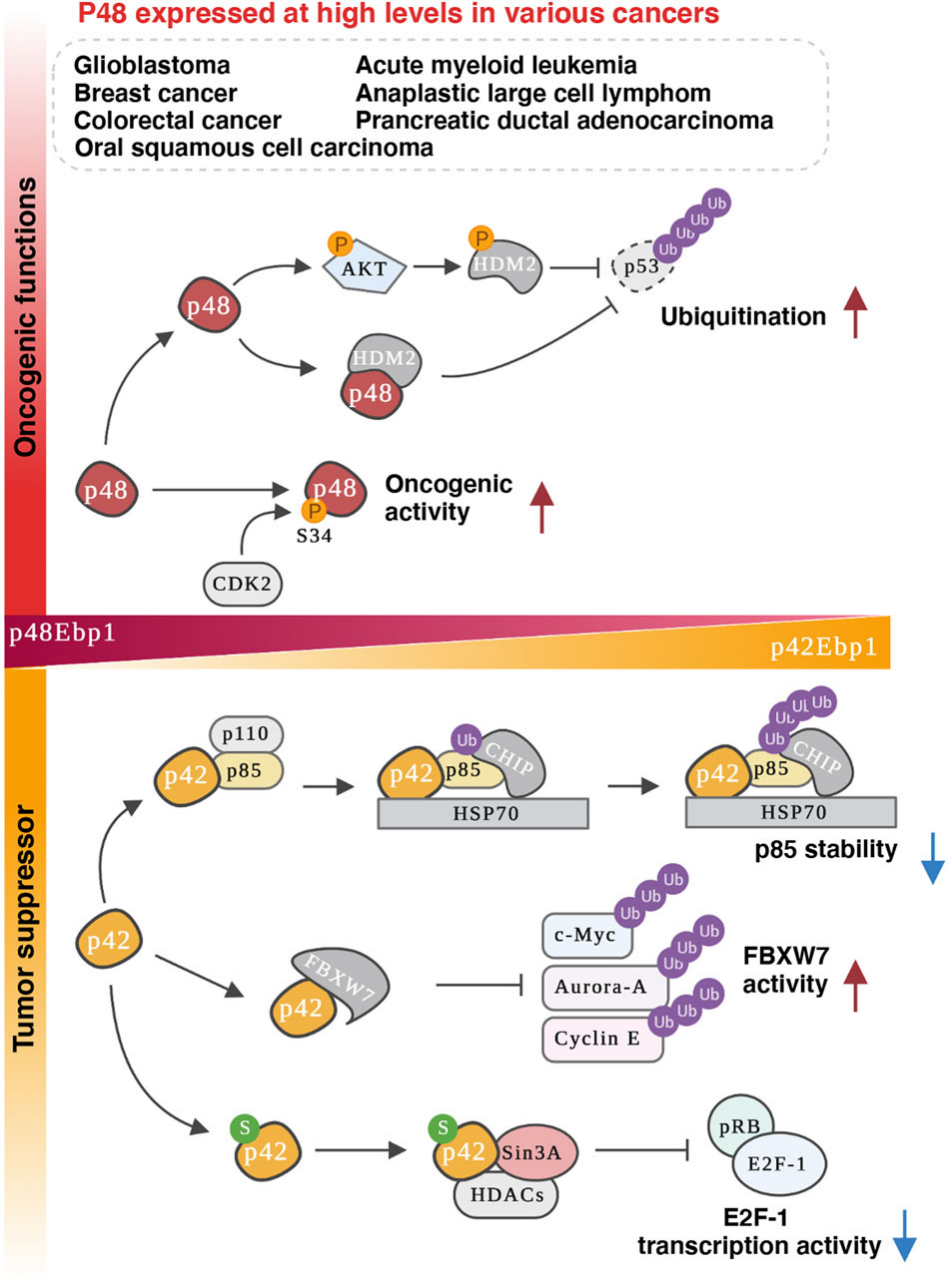
Distinct functions of EBP1 isoforms in cancer. Two EBP1 isoforms have different functions in various human cancers, along with differential binding partners. p48 (upper panel) is expressed at high levels in human patients with glioblastoma or breast cancer. Oncogenic effects of p48 result from, in part, downregulation of p53 protein levels by enhancing HDM2-mediated ubiquitination of p53 and subsequent degradation of p53 in cancer cells. In addition, CDK2-dependent phosphorylation of N-terminal S34 on p48 is associated with cancer development and progression. In contrast, p42 (lower panel) acts as a tumor suppressor, and its expression is extremely low in tumor cells, which is similar to that of other tumor suppressors. p42 inhibits PI3K activity, leading to HSP70/CHIP-mediated degradation of the p85 subunit of PI3K, thereby suppressing tumor growth. P42 also enhances the tumor suppressor function of FBXW7, increasing the association of FBXW7 and its target proteins, such as c-Myc, Aurora-A, or Cyclin E. Moreover, P42 binds to Sin3A and HDAC2, leading to repression of the transcriptional activity of E2F-1.

In our effort to identify the mechanism underlying the oncogenic activity of p48, we found that p48 overexpression in human glioma cells significantly stimulated the tumorigenic properties of cancer cells and enhanced tumor growth in mouse xenograft models. These oncogenic effects resulted from EBP1-dependent UPS regulation. P48 EBP1, and not p42 EBP1, binds to the E3 ligase domain of HDM2, which is famous for p53-mediated tumor suppression and the enhancement of the association of HDM2 and p53. The promotion of p53 poly-ubiquitination and the degradation leads to reduced levels and activity of p53. In addition to negatively regulating p53 by abrogating its interaction with HDM2, we proposed an alternative mechanism by which p48 negatively regulates p53. Specifically, we found that p48 stimulates Akt-mediated HDM2 phosphorylation, which leads to reduced protein levels of HDM2^6^. In addition, phosphorylated HDM2 is confined to the nucleus, and enhanced Akt activity prevents HDM2 self-ubiquitination^7^. In addition to inhibiting the tumor suppressor activity of p53, p48 exerts oncogenic activity through cyclin-dependent kinase 2 (CDK2)-dependent phosphorylation of its N-terminal domain at serine 34 residue, which is absent from p42. Mutating this phosphorylation site to alanine (S34A) prevents its phosphorylation. The loss of phosphorylation abrogates the ability of p48 to accelerate tumor cell growth both in vitro and in vivo, suggesting that CDK2-mediated phosphorylation of serine 34 of p48 is critical for the tumorigenic activity of p48 in human cancer cells^26^. The details of the protein domain and post-translational modifications involved in the functions of EBP1 isoforms are depicted in Fig. 2, and the current understanding of p48 EBP1- and p42 EBP1-mediated tumorigenesis is summarized in Fig. 3.

## The role of p48 EBP1 in development

Despite independent studies from the past 20 years that have demonstrated that EBP1 is an essential regulator of various cellular functions in cancers and in the development of mammalian cells, the precise molecular mechanisms of how EBP1 contributes to the multiple steps of cellular processes are not well defined. Few studies are available that provide in vivo evidence of EBP1 functions due to a lack of an appropriate mammalian model systems. We have previously demonstrated that p48 EBP1 is the major isoform in the developing brain neurons of rats and during murine brain development, whereas there is no detectable p42 EBP1 expression during the embryonic stage^4,27^. This also correlated with the ErbB3 expression profile, which is extremely limited in the developing CNS of mice. ErbB3 mRNA was not detectable during embryogenesis, but it started to be detected at P7^45^, which is coincident with expression of p42 EBP1 during the postnatal development period. These findings reflect that p48 EBP1 does not bind to ErbB3, and only p42 EBP1 is a binding to ErbB3, suggesting that p48 EBP1 could contribute to embryonic development independent of ErbB3.

Indeed, only p48 EBP1 was highly expressed during developmental myogenesis as well as regenerative myogenesis in mice, as it controlled the balance between proliferation and differentiation in the chick embryo model^17^. Moreover, in adult mice, EBP1 expression was undetectable in quiescent satellite cells and myogenic stem cells. However, it was induced during the activation of satellite cells. Furthermore, in chick embryos, specific depletion of EBP1 inhibited muscle progenitor differentiation, indicating a conserved function of EBP1 in the regulation of embryonic muscle progenitors and adult stem cells^17^. In addition, Ebp1 mRNA levels were increased from E9 to E20 in chicken embryonic muscle. Moreover, in cultured myoblasts, Ebp1 was upregulated in proliferating cells and decreased at the early stage of myogenic differentiation, revealing that EBP1 contributes to myoblast differentiation by inhibiting SMAD2/3 signaling^46^. In flies, overexpression of EBP1 disrupts muscle progenitors, leading to neurogenic-like states^47^.

A recent elaborate study from^18^ suggested that EBP1 binds to the homeodomain-containing transcription factor Six1 protein and affects the formation of the complex containing Six1 with its cofactor Eya1 in HEK293 cells. In a *Xenopus* fibroblast-like cell line (XTC-2), EBP1 activates gene transcription and modestly modulates the transcriptional activity of the Six1-Eya1 complex. In contrast, in HEK293 cells, EBP1 represses transcription of both an artificial and a natural reporter, revealing that EBP1 might regulate gene expression either positively or negatively, depending on cell context. The authors also demonstrated that overexpression of p48 EBP1 does not affect proliferation, whereas its reduction elicits reduced proliferation and apoptosis in the *Xenopus* embryo. In healthy cells, p48 EBP1 maintains cell growth and survival and has balanced expression with its isoforms, which is postulated not to be the case in cancer cells. EBP1 contributes to craniofacial development for proper formation of the neural border zone and its cranial derivatives, neural crest, and cranial placode genes. While six1 is increased upon EBP1 knockdown in a mouse myoblast cell line^17^, six1 expression was regulated in an EBP1-dose-dependent manner in the preplacodal ectoderm of an embryo, indicating the differential role of EBP1 in myogenesis and craniofacial development. This may be due to different binding partners or differential transcriptional regulatory functions of EBP1 in specific cellular contexts.

In human embryonic stem cells, only p48 EBP1 interacts with developmental pluripotency-associated 4 (DPPA4), and this interaction is reduced upon differentiation of pluripotent cells^16^. However, DPPA4 is associated with transcriptionally active chromatin along with both phosphorylated RNA polymerase II and H3K4 trimethylation in mouse ESCs^48^, Knoepfler and his colleagues have shown that DPPA4 has transcriptional repression activity^49^. They reported that EBP1 inhibits the transcriptional repression of DPPA4, thereby enhancing stem cell-related transcriptional regulation at specific genomic loci or cooperating in activating transcription at native targets^16^.

As the *Ebp1* knockout mouse model was not available, a previously^50^ generated gene trap of the *Ebp1* gene was generated via an insertion at intron 2, and the homozygous *Ebp1*^(−/−)^ mice were viable but were 30% smaller than wild-type littermates and exhibited transient growth retardation. For more than 10 years, no other report was presented using that model. In our study, we generated conditional deletion of *Ebp1* exons 6–10 to avoid targeting *Ebp1* exon 1 because the short distance between the *Ebp1* and *Erbb3* genes (~1.3 kb) could lead to the deregulation of *ErbB3* expression and affect *ErbB3* putative regulatory elements present in the 3′ region of the *ErbB3* gene^19^. Unlike the only previous report of *Ebp1*-deficient mice carrying a gene trap insertion that showed that *Ebp1* knockout mice were viable^50^, our *Ebp1* knockout mouse model resulted in embryonic death between E13.5 and E15.5. Moreover, knockout embryos exhibited prominently dilated vessels and hemorrhages throughout the entire body, particularly in the brain, and they displayed dilated cartilage primordium and deficient brain organogenesis as well as having a much smaller body and whole-brain volume compared with those of the control mice.

Moreover, heterozygous mice appeared to be smaller (~28% smaller at 1 week), yet they were able to gain bodyweight comparable to that of *Ebp1*^(+/+)^ WT mice after 2 months. Genetic ablation of *Ebp1* causes massive cell death and dysregulation of the epigenetic repression unit SUV39H1/DNMT1. *Ebp1*^(−/−)^ mice demonstrated the upregulation of Suv39H1-dependent H3K9 trimethylation and activation of DNMT1, revealing markedly increased global DNA methylation. EBP1 binds directly to the promoter region of DNNT1 and represses its transcription.

**Fig. 4:**
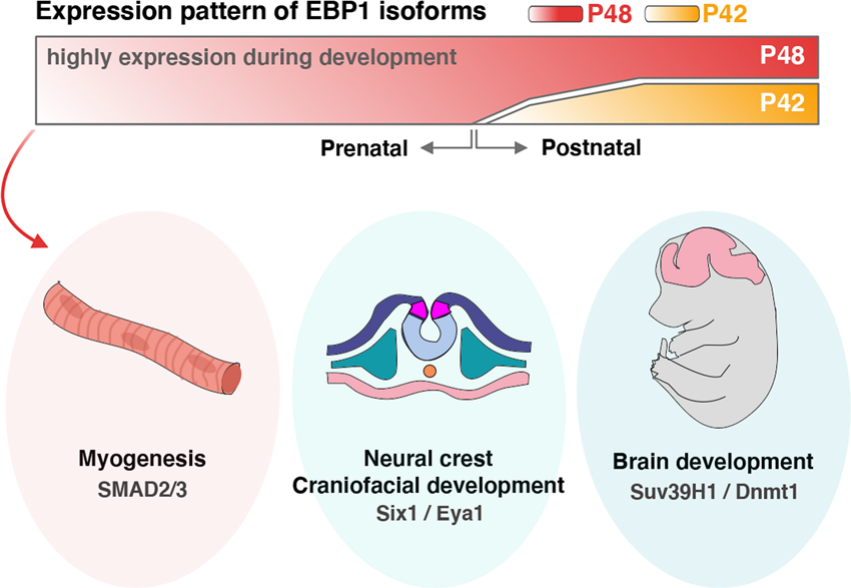
Roles of EBP1 isoforms in development. The two isoforms of EBP1 have a different expression patterns. During development, p48 is highly expressed as a major isoform contributing to myogenesis, neural crest and craniofacial development and brain development. p42, however, is expressed after the postnatal period, and its role in development has not yet been reported.

On the other hand, EBP1 interferes with DNMT1-mediated DNA methylation on its target gene; thus, the *Survivin* promoter enhances gene expression. Hence, we highlight in vivo evidence for an essential role of EBP1 in epigenetic regulation during embryonic development^19^ (Fig. 4). The next challenge will be the investigation of EBP1 on developmental disorders in humans involved in epigenetic diseases and the regulation of EBP1 expression during development.

## The role of EBP1 in the regulation of transcription and translation

**Fig. 5:**
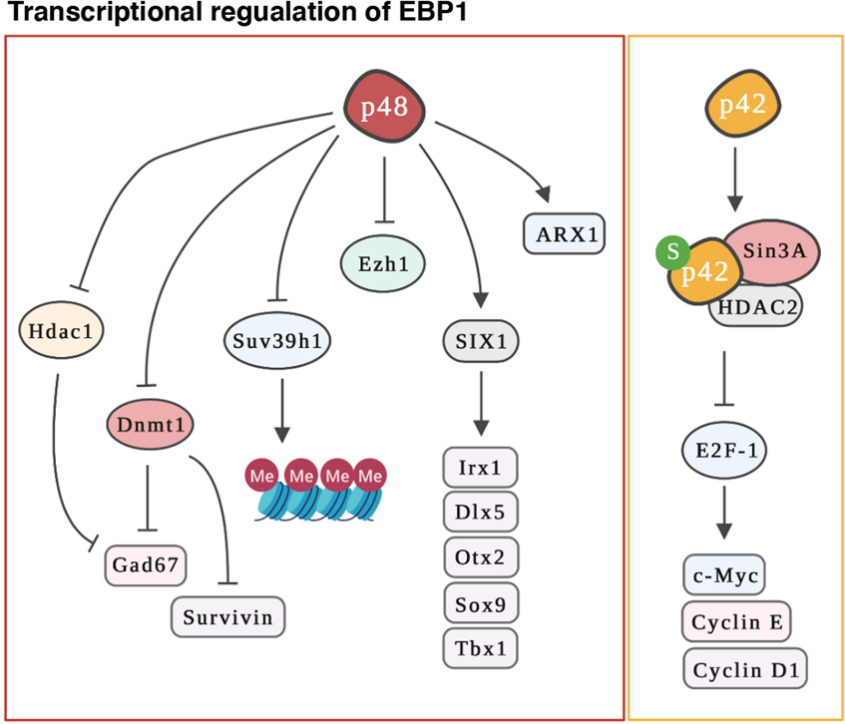
Transcriptional regulation of EBP1. P48 represses the transcription of epigenetic regulators such as Hdac1, Dnmt1, Suv39H1, or Ezh1, whereas it enhances the transcriptional activity of SIX1 or ARX1. In particular, P48 directly binds to the promoter region of Dnmt1 or Hdac1 to inhibit their transcription as well as the expression of target genes, such as Survivin or Gad67, during brain development in mice. P42 interacts with the Sin3A/HDAC2 complex and suppresses the transcription of E2F-1-mediated genes such as c-Myc, Cyclin E, or Cyclin D1.

Emerging evidence highlights that EBP1 can modulate transcriptional activity in many types of cancer and stem cells^3,16^ (Fig. 5). p42 EBP1 localizes predominantly to the cytoplasm and translocates to the nucleus upon growth factor stimulation, while p48 is distributed in the cytoplasm and the nucleus/nucleolus^2,14^. The differential cellular localization of EBP1 isoforms enables distinct regulatory functions in gene transcription. p42 EBP1(assuming as following by the employed plasmid) revealed that it represses the transcription of E2F1-mediated genes (cyclin E, cyclin D1, and c-myc), in addition to E2F1^29,32^, through recruitment of HDAC2 or through interaction with retinoblastoma (Rb). However, p42 EBP1 still inhibits the transcription of cyclin E Rb-null cells. This leaves questions to be answered regarding a specific network of p42 EBP1 in the repression for E2F-mediated transcription^32^. p42 EBP1 has also been shown to suppress androgen-mediated gene transcription, tumorigenesis of prostate cancer cells and metastasis of salivary adenoid carcinoma cells in mice^32,36,51^. However, due to the limitation of p42 EBP1 expression during the prenatal period, it is not yet known what the exact role is of p42 EBP1 in the embryonic development of mice or in brain development.

In addition to cancer cells, the developing hippocampal neurons from rats also exhibits strong association of p42 EBP1 with Rb, while p48 EBP1, especially the S360 phosphorylated form, barely binds to Rb. It is thought this may allow tight interactions between E2F1 and Rb, which thereby lead to E2F1 inactivation and cell cycle arrest in the G1 phase^27^, which account for the loss of further cell proliferation in neurons. In addition, EBP1 activates gene transcription in XTC-2 cells, whereas p48 EBP1 represses transcription in HEK293 cells, indicating that transcriptional regulation of p48 EBP1 is dependent in the cellular context^18^. Moreover, in a *Xenopus* embryo, p48 EBP1 acts as a corepressor of the six1 transcription factor and is required for neural crest and optic development. Furthermore, p48 EBP1 represses the transcriptional repression of DPPA4 in a pluripotent cell-specific context^16^. EBP1 suppressed HNF4a (hepatocyte nuclear factor 4a)-mediated transcription of its target genes that are implicated in insulin secretion in pancreatic β-cells through binding to HNF4a in the same binding pocket as other well-known coactivators, such as SRC1 or PGC1a^52^.

Employing conventional *Ebp1*^(−/−)^ mouse embryos and conditional homozygous mutant [Nestin-Cre; *Ebp1*^*flox/flox*^] analysis, we have shown that *Ebp1* expression is negatively correlated with genes associated with transcriptional repression, such as *Dmnt1, Suv39H1, Ezh1*, or *Prdm5, w*hereas a positive correlation was seen with the transcription activation-related gene *Dot1*. Moreover, we found higher levels of H3K9 trimethylation in *Ebp1*^(−/−)^ MEFs than in WT MEFs and a lack of EBP1 association with heterochromatin. In particular, p48 EBP1 directly binds to the Dnmt1 promoter region and represses Dnmt1 expression, thereby relieving Dnmt1 target genes from promoter methylation. In addition, p48 EBP1 competes with Dnmt1 for target gene DNA binding, disrupting Dnmt1-mediated promoter methylation. Collectively, our study suggests that EBP1 is a potent regulator of gene expression that may influence the regulation of multiple genes by modulating epigenetic regulators during development^19^. Intriguingly, in postnatal heterozygous *Ebp1*^(+/−)^ mice, we found reduced hippocampal volume and mRNA levels of GAD67, which are both popular features of schizophrenia (SZ). *Ebp1*^(+/−)^ mice also showed SZ-like behaviors. Accordingly, both Dnmt1 and EBP1 bind to the promoter region of histone deacetylase 1 (HDAC1), which leads to histone deacetylation of GAD67 and suppresses HDAC1 expression. Thus, the loss of EBP1 can facilitate the development of cognitive and social impairments in SZ patients via dysregulation of epigenetic repressors and reduction of GAD67 expression in the hippocampus^53^. Further investigation is required for the dissection of the epigenetic machinery in various regions of the brain and developmental and neurological diseases. Such studies may inform therapeutic strategies to restore the balance of gene expression.

In addition to its role in controlling transcription, EBP1 has been thought to be involved in protein synthesis, as EBP1 interacts with an array of RNA targets, including FMDV-IRES^12^, the 3′-untranslated region of bcl2 mRA^13^, rRNA^14^ and ss/ds RNA^10^. EBP1 has also been implicated in ribosome biogenesis via interaction with NPM^9^. In NPM1-mutated AML, HOXB-AS3, a lncRNA embedded in the HOXB locus, binds to p48 EBP1 and guides it to rDNA, where it augments the interaction of EBP1 with NPM in the nucleolus, ultimately contributing to efficient protein translation^54^. In primary T cells, p48 EBP1 is involved in rRNA synthesis by regulating TIF1A^55^.

Considering the structural similarity of p48 EBP1 with methionine aminopeptidase (MetAP), which cleaves the first methionine from the growing polypeptide chain, EBP1 lacks catalytic activity. It is not surprising to find EBP1 in complex with ribosomes. EBP1 has been identified as the human homolog of the 60S ribosomal nuclear export factor Arx1 from *Saccharomyces cerevisiae*, yet no evidence for functional homology has been reported^56^. However, a functional disconnect between the homologs would not surprising as EBP1 does not possess structural features that are important for ribosome biogenesis factor interaction in yeast Arx1, and it does not bind to nucleoporins that are required for pre-60S nuclear export factor^57,58^. In particular, EBP1 binds to nontranslating human 80S ribosome, competing with MetAP and blocking exit from the ribosome because of significant interactions with general uL23/uL29 docking sites for nascent chain-associated factors. However, it is not known whether EBP1 binding arrests protein synthesis. The precise role of two isoforms of EBP1 in translational regulation and molecular mechanisms underlying specific cellular contexts pathologically and physiologically will provide insight into the contribution of EBP1 in various cellular functions and diseases.

## Concluding remarks

As we state in this manuscript, the two EBP1 isoforms perform different functions in many aspects of cellular events, including cell proliferation and survival, regulation of gene expression, and protein synthesis in the development of the disease. Many questions regarding isoform specificity in cells remain unanswered and have raised confusion and/or controversial results. The two isoforms have different spatial/temporal expression patterns; during embryogenesis, only p48 EBP1 is expressed, and p48 EBP1 is dominantly expressed in cancers and many types of cell lines, which is in agreement with the protein instability of p42 EBP1, as mediated by UPS, and our experience in the difficulty of detecting endogenous p42 EBP1.

These isoforms interact with a variety of signaling molecules, transcriptional/translational machinery proteins, and UPS proteins. While they share some binding partners, they also have specific binding partners. For example, both isoforms of EBP1 can interact with NPM to regulate cell cycle progression^9^. Through the N-terminal domain, which is absent in p42 EBP1, p48 EBP1 interacts specifically with HDM2 for p53 degradation and binds to CDK4 to mediate S34 phosphorylation^6,26^. Interestingly, p42 EBP1 specifically interacts with the p85 subunit of PI3K and HSP70/CHIP E3 ligase complex through the C-terminal domain, which is present in both p42 and p48 EBP1^7^. A balanced expression of two isoforms may be essential for maintaining normal cellular growth, as both EBP1 isoforms are involved in the linkage of ubiquitin to the target of E3 ligases.

It will be important to elucidate whether the two isoforms distinctively bind to epigenetic factors or transcriptional regulators in different contexts. In particular, isoform specificity may be relevant to transcriptional regulation or translational regulation, as EBP1 demonstrated both transcriptional repression and activation function^18^. Moreover, no apparent translational inhibitory effect of p48 EBP1 or the association of p42 EBP1 with the 80S ribosome has been determined^15^. For instance, during development, the uniquely expressed p48 EBP1 binds to the Dnmt1 or survivin promoters and may coordinate with the regulation of multiple genes by modulating epigenetic regulators^19^. However, we do not yet know in the case of cancers or other developmental/neurologic disease conditions, whether the two isoforms are differentially expressed and/or are altered in DNA binding ability by binding partners. To date, only a few studies have investigated in detail the transcriptional/translational regulation of two EBP1 isoforms. However, future studies will likely reveal critically important functions for EBP1 isoforms in development and human diseases.
